# Preparation of Iron-Based Sulfides and Their Applications in Biomedical Fields

**DOI:** 10.3390/biomimetics8020177

**Published:** 2023-04-24

**Authors:** Yefan Duan, Jianfei Sun

**Affiliations:** State Key Laboratory of Bioelectronics, Jiangsu Key Laboratory of Biomaterials and Devices, School of Biological Science and Medical Engineering, Southeast University, Nanjing 210009, China; yefanduan@foxmail.com

**Keywords:** iron-based sulfides, iron sulfide clusters, Fenton reaction, biomedical application

## Abstract

Recently, iron-based sulfides, including iron sulfide minerals and biological iron sulfide clusters, have attracted widespread interest, owing to their excellent biocompatibility and multi-functionality in biomedical applications. As such, controlled synthesized iron sulfide nanomaterials with elaborate designs, enhanced functionality and unique electronic structures show numerous advantages. Furthermore, iron sulfide clusters produced through biological metabolism are thought to possess magnetic properties and play a crucial role in balancing the concentration of iron in cells, thereby affecting ferroptosis processes. The electrons in the Fenton reaction constantly transfer between Fe^2+^ and Fe^3+^, participating in the production and reaction process of reactive oxygen species (ROS). This mechanism is considered to confer advantages in various biomedical fields such as the antibacterial field, tumor treatment, biosensing and the treatment of neurodegenerative diseases. Thus, we aim to systematically introduce recent advances in common iron-based sulfides.

## 1. Introduction

There is a significant trend in developing the next generation of materials that are suitable for practical clinical needs. Different from the new energy, optoelectronic device and electrochemical fields, the biomedical field not only requires materials with reasonable physical, chemical, electronic and optical properties, but also pays more attention to higher biological safety, to facilitate implantation or injection into the human body [1,2,3]. The size of materials used for injection will be strictly controlled to avoid long-term toxicity in the body due to low renal clearance, which will result in impaired liver and kidney function [4,5,6]. The diversity of the internal microenvironment and application scenarios therefore brings more challenges to nanomaterials. As a result, it is of great importance to develop and optimize the performance of materials to support multi-function platforms with excellent biocompatibility in the biomedical field. 

Superparamagnetic iron oxide nanoparticles (SPIONs) were the first generation of nanomaterials to achieve accurate clinical applications. They are approved by the United States Food and Drug Administration (FDA) as a contrast agent for magnetic resonance imaging (MRI) due to their low toxicity, biocompatibility, and biodegradability, which implies that iron-based materials have potential clinical practicability [7,8,9,10,11]. Subsequently, iron-based sulfides came into view as a type of iron-based material, and gained a lot of interest as a potential object that can be developed and used in biomedical applications. Iron-based sulfides have an enormous variety of compositions involving Fe_1-x_S [12,13,14], FeS [15,16,17], FeS_2_ [18,19,20], Fe_3_S_4_ [21,22,23] and some ternary compounds [24,25]. It is worth noting that the iron atoms in iron-sulfide compounds can bind to sulfhydryl ligands on cysteine in vivo to form a ferromagnetic and antiferromagnetic iron sulfide cluster [26,27]. In addition, sulfur atoms have 16 protons and 3 layers of extrinsic electrons, giving iron-based sulfides a more complex electronic structure, which further facilitates higher electron and charge transfer efficiency than iron-based oxides [28]. This also makes them more complex with varied properties, because physical and chemical properties often depend on the electronic structure. In contrast, iron-based sulfides can break through the opposition between reliable biosafety and superior multi-functionality, providing new opportunities for efficient biomedical applications.

Although the properties of iron-based sulfides are outstanding, they are still in an initial stage in terms of the biomedical field, and a comprehensive presentation of their properties is absent. Herein, we present the structure and properties of iron-based sulfides, consisting of iron sulfide minerals in nature and biological iron sulfide clusters in organisms. In the aspect of performance, the outstanding magnetic properties and catalytic activities are mainly described, and the structure is found to have a significant influence on them. Next, the preparation methods are presented based on two aspects: molecular crystal synthesis and biosynthesis. In addition, several biomedical applications based on electron transport mechanisms are proposed. Finally, the challenges and opportunities faced by iron-based sulfides in the biomedical field are subsequently clarified to boost a deeper understanding in the future.

## 2. Structure and Properties of Iron-Based Sulfides

### 2.1. Structure of Iron-Based Sulfides

Iron is a widely distributed element in nature, being found in soil particles, rocks, water sediments and living organisms [29]. Under specific temperatures and pressures, a chemical reaction will occur between iron and oxygen or sulfur to form natural minerals (iron oxide minerals and iron sulfide minerals). In organisms, iron ions are prone to combine with the cysteine residues of proteins, which may be followed by the formation of iron sulfide clusters. Herein, the common natural iron sulfide minerals include pyrite, pyrrhotite, marcasite and mackinawite. Table 1 shows their crystal structures. The basic structural units of iron sulfide clusters in living organisms are also mentioned.

Pyrite, the most abundant iron-based sulfide with FeS_2_ as the main component, and its crystal structure are attributed to the cubic system, in which there are one iron atom and six nearby sulfur atoms combined in an octahedral configuration with identical Fe–S bond distances. Each S atom consists of three iron atoms, and another S atom in a tetrahedral structure [30]. The length of the Fe–S bond is 2.26 Å, and the length of the S–S bond is 2.15 Å. Overall, FeS_2_ can be viewed as consisting of Fe^2+^ and S_2_^2−^ in a cubic symmetry with a slight distortion [31]. The band gap energy of bulk pyrite is 0.95 eV, and the optical absorption coefficient (5 × 10^5^ cm^−1^) is high [32]; thus, pyrite has been found to exhibit semiconducting properties.

Based on the fragile character of the S–S bond of pyrite, the sulfur atoms can be quickly released, resulting in the formation of another mineral named pyrrhotite [33]. The chemical formula of pyrrhotite is usually written as Fe_1−x_S (x ranges from 0 to 0.125), followed by a distinct Fe/S ratio, which results in a higher amount of S compared with FeS [34]. Thus far, four substances with near stoichiometric Fe/S ratios have been explored: Fe_7_S_8_, Fe_9_S_10_, Fe_10_S_11_ and Fe_11_S_12_. Among them, Fe_7_S_8_ and Fe_9_S_10_ are the two representative substances with various crystal structures, corresponding to monoclinic and hexagonal structures, respectively [35]. Consequently, the coordination modes between Fe and S atoms are vividly diverse. 

The main chemical component of marcasite is the same as that of pyrite (FeS_2_), but its crystal structure varies. Marcasite is in an orthogonal system, which is distinguished by edge-sharing FeS_6_ units along the c-axis of the unit cell and corner-sharing edges in other directions [36]. Regardless of whether pyrite or marcasite is described as trigonally distorted FeS6 octahedra and tetrahedrally coordinated S atoms, the S atom is coordinated to three Fe atoms and one other S atom, and it is worth noting that the property of marcasite is considered less stable than that of pyrite. Due to the similar characteristics between pyrite and marcasite in some crystallization directions, they can converge into each other under certain conditions. Recently, studies based on theoretical investigations revealed that the band gap of marcasite is around 0.8–1.1 eV, which is similar to that of pyrite [37]. These findings demonstrate that pyrite and marcasite possess semiconductor properties that have potential applications in transistors and electronic devices. 

Mackinawite is a layered iron sulfide mineral with a tetragonal structure, in which the Fe ion has +2 valence and the S ion has −2 valence [38,39]. It is a highly active precursor of other stable iron sulfide minerals (pyrite or marcasite). In the crystal structure, sulfur atoms and iron atoms exhibit tetrahedral coordination, connected by covalent bonds. Therefore, the structure of mackinawite is tight and stable. However, whether tetrahedral FeS is classified as a metal or a semiconductor phase has been a controversial issue. This dilemma was resolved when Huang’s team prepared tetrahedral FeS with excellent crystallization and stability under ordinary conditions, where the superconductivity was observed to be below 5 K for the first time in tetrahedral FeS, strongly suggesting that FeS behaves as a paramagnetic metal phase in the normal state [17]. In general, due to the abundant valence of the Fe atom and its lively properties, iron sulfide minerals exist in various forms and are often accompanied by the doping of other substances, which can be applied in multiple fields. 

**Figure 1:**
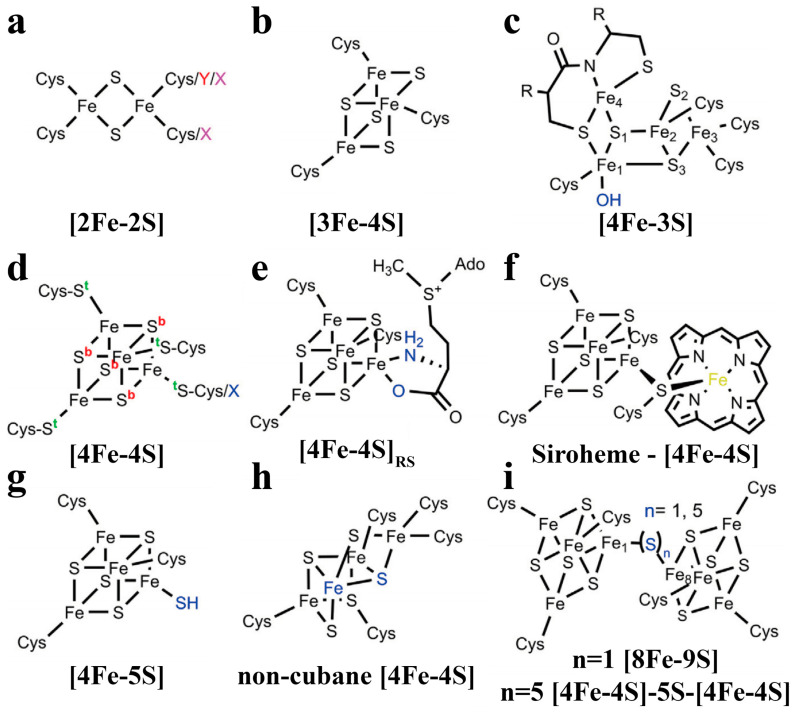
Common forms of [FeS] clusters. These clusters have various functional roles in different proteins. (**a**) [2Fe-2S] cluster; (**b**) [3Fe-4S] cluster; (**c**) [4Fe-3S] cluster; (**d**) [4Fe-4S] cluster; (**e**) [4Fe-4S]_RS_ cluster; (**f**) siroheme-[4Fe-4S] cluster; (**g**) [4Fe-5S] cluster; (**h**) non-cubane [4Fe-4S] cluster and (**i**) [4Fe-4S]-S_n_-[4Fe-4S] clusters (n = 1, 5) [46]. Copyright 2022 Elsevier.

In addition to iron-based sulfides in mineral form occurring during geological processes, the iron element is also widely distributed in organisms in the form of iron sulfide clusters, as the oldest classes of bioinorganic cofactors. From the perspective of structure, the Fe and S atoms exist in various stoichiometries, and the coordinating ligands of the irons in the cluster are different, in which cysteine generally completes tetrahedral S coordination at each Fe site [40]. [2Fe-2S] [41] and [4Fe-4S] [42] are the most generic common stoichiometries of biological iron sulfide clusters, and some other patterns, such as [3Fe-4S] [43], [4Fe-3S] [44], [8Fe-7S] [45] and [4Fe-5S] [46], have also be explored with the development of research (Figure 1). Conversion reactions have existed in homogeneous or heterogeneous systems of initial or intermediate clusters. Iron sulfur clusters readily accept or provide a single electron, participating in complex chemical reactions in which two [2Fe-2S] can transform into one [4Fe-4S] ([2Fe-2S]^2+, 1+^ 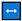 [4Fe-4S]^2+^), and the process is reversible [47]. dimerization based on electron transfer exists universally in iron sulfide clusters of disparate structures, enriching the forms and types of iron sulfide clusters to satisfy the functional requirements of specific biological scenarios. On account of the conspicuous structural plasticity and versatile electronic characteristics, iron sulfide clusters play an important role in electron transfer, biological catalysis, ATP production, regulation of gene expression and synthesis of proteins [48]. 

### 2.2. Properties of Iron-Based Sulfides

Iron, as a very active element in group VIII transition metals, has various valences such as 0, +2 and +3, and possesses a face-centered cubic unit cell or hexagonal lattice structure proximately to metal elements (Pt, Pd, Mo, W, Ni, Co, etc.) owning traditional catalytic activity. Moreover, its d-atomic orbital is not complete, leading to an instinctively catalytic activity of iron-based sulfides [49]. One prominent catalytic property is based on the Fenton reaction [50], where reductive Fe^2+^ catalyzes H_2_O_2_ to produce potent oxidizing hydroxyl radicals (·OH). This performance of iron-based sulfides has been extensively applied in wastewater treatment [51], cancer catalytic therapy [52] and antibacterial therapy [53]. On top of that, hematite is a typical magnetic mineral in nature, and the S element is one of the oxygen group elements, so some iron-based sulfides have magnetic properties, including pyrrhotite (Fe_1−x_S) and melnikovite (Fe_3_S_4_). Interestingly, biological iron sulfide clusters also have magnetic properties in vivo [54]. These results indicate that iron sulfide clusters can not only participate in energy metabolism and the enzymatic reactions of cells but can also be developed as magnetic receptor proteins to gift organisms with magnetic sensitivity. The Fenton reaction was first presented by Henry J. Fenton, subsequently drawing significant interest in the field of wastewater treatment [55,56]. During the process of the reaction, potent oxidative hydroxyl radicals occur in the presence of Fe^2+^ and H_2_O_2_, which play an essential role in degrading water pollution. Chemodynamic therapy (CDT) is an efficient strategy for cancer treatment inspired by the Fenton reaction to generate highly cytotoxic ·OH [57]. In cancer cells, H_2_O_2_ is an overproduced non-radical reactive oxygen species, which can easily diffuse across biological membranes. If iron exists, H_2_O_2_ will be converted to •OH locally according to the Fenton reaction and further damage the biological system including lipids, DNA and amino acids in proteins [58]. Meanwhile, the accumulation of •OH will drastically exacerbate oxidative stress in cells and subsequently induce cell death. Hence, significant attention has been paid to Fenton catalysts such as iron minerals, including FeS_2_ [59], FeS [60] and Fe_1-x_S [13]. However, the process of the Fenton reaction is complicated and constituted of a chain of reactions. As shown in Reaction 1, •OH is firstly generated based on H_2_O_2_ and Fe^2+^. Then, the produced Fe^3+^ is reduced by H_2_O_2_ and the reproduced Fe^2+^ (Reaction (2)), involving a circular process. The continuous operation of this cycle is rigorous and requires large amounts of H_2_O_2_ and an optimal pH range (pH = 3.0–4.0). Nevertheless, if the pH is above 3.0, Fe^3+^ can easily convert into inactive Fe(OH)_3_ precipitation and significantly block further reactions. Therefore, the heterogeneous Fenton reaction has been proposed, in which •OH is continuously generated, avoiding the precipitation of Fe(OH)_3_. Besides the reaction between Fe ions and H_2_O_2_ (Reactions (1) and (2)), the heterogeneous Fenton reaction concerns the electron exchange between surface Fe and H_2_O_2_ (Reactions (3) and (4)). It is worth noting that the efficiency of the Fenton reaction will be decreased in the presence of Fe^3+^ or Fe(Ⅲ) reduction, because of its lower rate constant (0.001–0.01 M^−1^s^−1^) [61]. Therefore, it is a reliable method to excite the reaction system by reducing and consuming Fe^3+^ [62]. Additionally, the activity of the Fenton reaction is related to the temperature, time, pH, concentration, surface morphology and stability of substrates. Most reactions are limited by a narrow pH range (pH = 3.0–5.0) and accompanied by limited kinetics, poor permeability, the short half-life of hydroxyl radicals and other deficiencies [63]. To overcome these deficiencies, materials need to be systematically designed, including regulating their morphology and electron structure at the micro level, to provide more active sites to effectively enhance the Fenton catalytic performance.

Fe^2+^ + H_2_O_2_ → Fe^3+^ +·OH + OH^−^ Reaction (1)

Fe^3+^ + H_2_O_2_ → Fe^2+^ + HO_2_· + H^+^ Reaction (2)

≡Fe(Ⅲ) + H_2_O_2_ → ≡Fe(Ⅱ) + HO_2_·+ H^+^ Reaction (3)

≡Fe(Ⅱ) + H_2_O_2_ → ≡Fe(Ⅲ) + HO· + OH^−^ Reaction (4)

**Figure 2:**
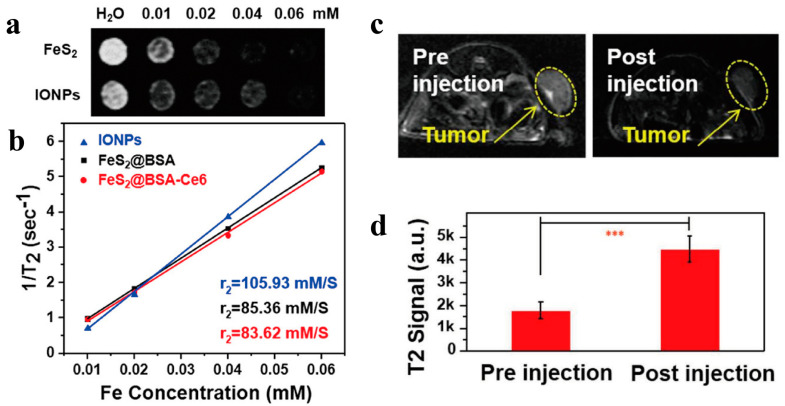
T2-weighted MRI performance of FeS_2_@BSA-Ce6 nanodots. (**a**) MR images of FeS_2_@BSA nanodots and IONPs; (**b**) relative relaxation rate R_2_ of FeS_2_@BSA nanodots, FeS_2_@BSA-Ce6 nanocomplex and iron oxide nanoparticles (IONPs); (**c**) MR images and (**d**) quantified MR signals of 4T1 tumor-bearing nude mice before and 8 h after iv injection of FeS_2_@BSA-Ce6 nanodots [69]. Means ± SD are shown (n = 3) (*** *p* < 0.001) Copyright 2017 American Chemical Society.

The magnetic properties of iron-based sulfides have been intensely investigated for numerous applications, such as magnetic resonance imaging contrast agents, magnetothermal therapy, magnetically responsive photodetectors and magnetically responsive biosensors. It is well known that iron oxide nanoparticles have excellent magnetic properties. Parts of iron-based sulfides have also been found to have magnetic properties, and the size, morphology and composition of the materials will affect the performance of the magnetic properties. Moreover, crystallinity is positively correlated with magnetic properties [7]. Clinically, MRI contrast agents can be divided into longitudinal relaxation contrast agents (T1) and transverse relaxation contrast agents (T2). In Table 2, the magnetic properties of various iron-based sulfides are presented. As a whole, the transverse relaxation rate (R_2_) of iron-based sulfides is much better than the longitudinal relaxation rate (R_1_), so they are mainly used in T2-weighted magnetic resonance imaging. Liu’s group developed strong superparamagnetic FeS nanoplates using a simple one-step method, in which the transverse relaxation rate of magnetic resonance (R_2_) was 209.8 mM^−1^ S^−1^, much higher than most of the T2 contrast agents that have been used clinically (105.93 mM^−1^ S^−1^ for Fe_2_O_3_ nanoparticles, 72 mM^−1^ S^−1^ for ferumoxsil, 151 mM^−1^ S^−1^ for ferrixan, 98.3 mM^−1^ S^−1^ for ferumoxide) [64]. This suggests that iron-based sulfides may be a promising class of magnetic nanomaterials with potential prospects for future clinical translation. Subsequently, different types of iron-based sulfides such as Fe_1−x_S, FeS_2_, Fe_3_S_4_ and some heterostructures containing iron sulfides have been developed to satisfy the requirements of the specific application (Figure 2).

In addition, as a type of iron sulfide cluster binding protein, each protein monomer binds an iron sulfide cluster in the form of a ferro-disulfide, which has been found to have potential magnetic properties and proved to be related to the magnetic sensing ability of some species, becoming a breakthrough in the field of biological magnetic sensing. Xie’s group proposed a protein-based biocompass model, which indicated the existence of an iron sulfide protein called the magnetoreceptor (MagR), which is assembled via linear polymerization to form a rod-like protein complex (magnetosensor). Like a small magnetic bar, it has a north and south pole. It was observed under an electron microscope that the MagR protein complex could sense the weak magnetic field of the earth, and it had an obvious intrinsic magnetic moment [56,65,66,67]. Another interesting finding is that transfection of clMagR/clCry4 stimulated the formation of iron oxide nanoparticles in the presence of Fe^3+^, and further impacted MRI T2 contrast properties [68]. Based on this, further research needs to be carried out to reveal the mysterious mechanism of animal magnetic induction and biological navigation, which will be a promising boost in the development of a new generation of bionic navigation and magnetic induction devices.

## 3. Synthetic Methods of Iron-Based Sulfides

Molecular crystal synthesis and biosynthesis are both important methods for the synthesis of iron-based sulfides, but there are significant differences in the objects and principles. Molecular crystal synthesis refers to the study of how to prepare and construct molecular structures with specific structures and properties, focusing on the exploration of reaction principles and reaction conditions. Biosynthesis focuses on the synthesis of molecules in living organisms, involving energy transfer, electron transfer and enzymatic kinetics. The synthesis of iron-based sulfides will be elaborated from these two dimensions. 

### 3.1. Molecular Crystal Synthesis

The typical molecular crystal synthesis of iron-based sulfides includes the chemical vapor deposition method, hydrothermal synthesis, chemical coprecipitation and high-temperature pyrolysis. Among the above methods, common iron sources that can be used in the laboratory are FeCl_2_·4H_2_O, Fe(NO_3_)_3_·9H_2_O, FeSO_4_, etc., and usual sulfur sources include sodium thiosulphate (Na_2_S_2_O_3_), sulfur powder (S), sodium sulfide (Na_2_S) and thiourea (NH_2_CSNH_2_). The time, the temperature during the reaction and the proportions of the iron and sulfur sources have a significant influence on the composition and structure of the final crystals.

The chemical vapor deposition method is a standard method for preparing two-dimensional ultra-thin materials. Thus far, different types of two-dimensional iron-based sulfides have been attempted. For example, pure semiconductor-phase FeS_2_ films can be directly grown on the CoS_2_ substrate at high temperatures, and di-tert-butyl disulfide (TBDS) has been used as a novel sulfur precursor [75]. A single layer of high-quality tetragonal FeS films was prepared via in situ topological preparation by Shigekawa [76]. In addition, the direction of growth can be controlled by introducing a template. Regular hexagonal FeS_2_ crystals were grown at 600 °C based on the template of graphene [77]. Doping and phase regulation can be carried out using the CVD method to obtain two-dimensional materials with excellent electrical, optical, magnetic and mechanical properties. Although the growth mechanism based on chemical reactions is complex, many studies have shown that the reaction temperature, gas flow rate and molten salt all have an influence on the product and its performance.

Hydrothermal synthesis is a widely used method applied in laboratory and industrial production due to its advantages of a simple operation and high yield. The hydrothermal synthesis of FeS_2_ microspheres [78], metastable FeS_2_ [19], FeS_2_ quantum dots [79], FeS nanosheets [80], FeS nanodots [81], Fe_3_S_4_ microspheres [82], Fe_3_S_4_ nanoflowers [83] and other iron-based sulfides have been reported thus far. Melonie et al. synthesized highly crystalline FeS nanosheets via surfactant-assisted hydrothermal synthesis, and showed great control over the size, shape and thickness of the final product by changing the amount of the iron source and surfactant [84]. From previous studies, it can be summarized that surfactants such as cetyltrimethylammonium bromide (CTAB), cetyltrimethylammonium chloride (CTAC) and polyvinylpyrrolidone (PVP) will spontaneously form spherical or rod-like micelles attached to the surface of the crystal nucleus to restrict the growth of materials and affect the morphology and particle size of the product, when they disperse in the solvent [85]. If the template is introduced during the hydrothermal reaction, then the crystal nucleus would attach to the template and facilitate the growth of composite or heterogeneous structures. Inspired by this mechanism, many materials with composite structures have been created, such as rGO/FeS composite [86], FeS_2_@graphene [87], rGO/FeS_2_ hybrid microspheres [87] and Fe_3_S_4_ NPs@rGO [88].

The procedure of the solvothermal method is similar to that of hydrothermal synthesis, the most significant difference being the choice of solvent, and that the solvothermal method prefers to utilize organic solvents (N’N-dimethylformamide, methanol, ethylenediamine, oleylamine, polyethylene glycol, etc.) as the solvent. Under high temperatures and pressures, the nucleation and recrystallization processes occur. The structural and chemical defects of crystal growth can be improved by adjusting the factors that affect the crystal growth rate (temperature, pressure, time, solvent and gas atmosphere), which can adjust the morphology, size, pore size and function of the crystal [89]. FeS nanosheet [90], rhombic FeS_2_ [91], octahedral FeS_2_ [92], FeS_2_ nanoparticle [93] and magnetic Fe_3_S_4_ [94] have been created using this method and applied in some areas. However, this method is not green, and the biocompatibility of the product is demanding up to the standard in the biomedical field, so further modification steps need to be performed before application.

### 3.2. Biosynthesis

Recently, more attention has been focused on biosynthesis owing to its advantages of being inexpensive, environmentally friendly and energy-efficient. It is an old strategy to prepare complex and pharmacologically active natural products, replacing plant separation and extraction with a lower extraction efficiency. Notably, it is a method that has the potential to synthesize nanomaterials by employing living organisms as biofactories, having special advantages compared with traditional chemical methods [95]. Biosynthesized nanoparticles are endowed with excellent biocompatibility, water solubility and an ultrasmall size, which are desirable in the biomedical field and applicable in bioimaging, biosensing, drug delivery and treatment [96]. FeS nanoparticles are representative iron-based sulfides that can be predominantly biosynthesized through sulfate-reducing bacteria (SRB) in anoxic environments [97]. Nonetheless, little is known about the mechanism of this process, especially regarding the electron transfer capacity and enzyme kinetics in cells. In previous studies, evidence can be found that the production of FeS is related to the metabolic process of SRB, which reduces sulfate (SO_4_^2−^) as an electron acceptor to form S^2−^ [98]. However, which biological groups, signaling pathways and protein expressions are involved during the reaction remain unclear. Therefore, in-depth exploration of biological mechanisms is urgently needed, and will contribute to the green controllable synthesis of related materials on morphological and structural scales. 

Iron sulfide clusters are types of iron-based sulfides widely existing in organisms, which have important biological functions. Iron sulfide clusters play an important role in cellular processes such as electron transport, redox reactions and signal transduction. The biosynthetic pathway of iron sulfide clusters is not fully defined, but some studies have shown that the biosynthetic process involves the interaction and regulation of various enzymes, proteins and cofactors. In organisms, iron ions are easily oxidized, so special transporters must be used to transport iron ions to the synthetic iron sulfur cluster. For example, iron sulfur cluster assembly protein A (IscA) can collect free iron in cells and transfer it to the iron sulfur cluster assembly enzyme (IscU) for assembly. Finally, the assembly of the iron sulfur cluster is promoted and completed on the IscU scaffold protein, because the scaffold protein has high structural stability and iron sulfide cluster binding ability. In conclusion, the biosynthesis of iron sulfide clusters is a complex process that has not been described explicitly here. Further study will help to better understand the formation and function of iron sulfide clusters in organisms and provide a theoretical basis for the design and synthesis of new iron sulfide cluster structures.

## 4. Biomedical Applications Based on Electron Transport Mechanism

The Fenton reaction uses H_2_O_2_ as a chain propagation medium to mediate the conversion between Fe^2+^ and Fe^3+^ through an electron transfer reaction, resulting in the generation of ·OH. Based on this mechanism, it can be used for antibacterial agents, biosensor construction and chemodynamic therapy. In recent years, ferroptosis, a new, highly iron-dependent programmed cell death mechanism that differs from apoptosis or necrosis, has also been widely explored [99,100,101]. When iron-based nanoparticles enter cells, lipid peroxidation and elevated reactive oxygen species (ROS) levels will occur, followed by the ferroptosis of cells. Ferroptosis can therefore be seen as the result of damage to the electron transfer chain and can be used in the efficient treatment of tumors [102] and neurodegeneration [103]. Based on this, this section mainly summarizes biomedical applications based on electron transport mechanisms, including in the antibacterial field, tumor treatment, biosensing and degenerative neurological diseases.

### 4.1. Antibacterial Field

**Figure 3:**
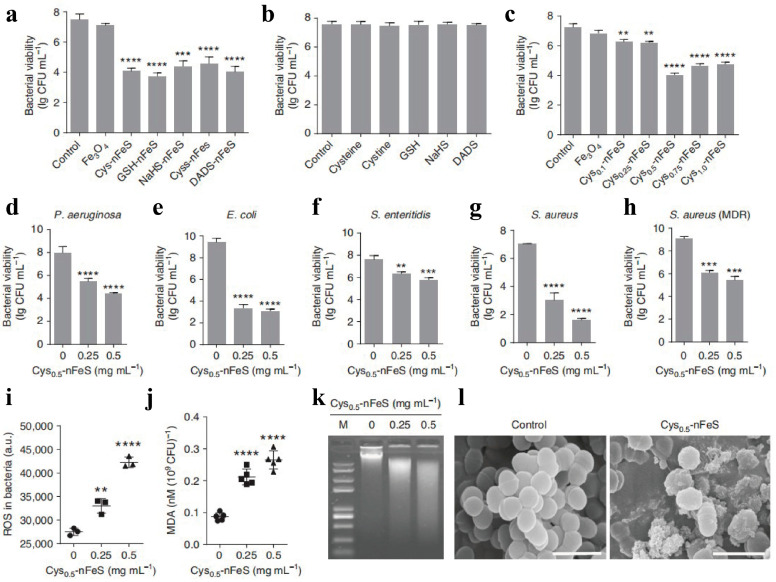
Antibacterial activity of nFeS. (**a**) Antibacterial activity of nFeS converted from different organosulfur sources against *S. mutans* UA159; (**b**) antibacterial (*S. mutans* UA159) activity of organosulfur compounds; (**c**) dependence of antibacterial (*S. mutans* UA159) efficacy of Cys-nFeS on the amount of cysteine input into the solvothermal synthesis; (**d**–**h**) antibacterial activity of nFeS on *P. aeruginosa*, *E. coli*, *S. enteritidis*, *S. aureus* and *S. aureus* (MDR), respectively; (**i**,**j**) ROS level and lipid peroxidation of bacteria treated with Cys-nFeS; (**k**) genomic DNA degradation of bacteria treated with Cys-nFeS; (**l**) SEM image of bacteria treated with Cys-nFeS [106]. Means ± SD are shown (n = 3) (** *p* < 0.01, *** *p* < 0.001, **** *p* < 0.0001) Copyright 2018 Nature Publishing Group.

The abuse of antibiotics leads to an increase in bacterial resistance, which is the dilemma faced by traditional antibacterial agents. Therefore, eliminating bacterial infection based on nanotechnology is a new solution [104]. Some inorganic metal elements naturally have good sterilization and inhibition of microbial reproduction, such as silver, copper and zinc. In addition, iron has also been found to have potential antibacterial ability. For example, iron oxide nanoparticles have peroxidase activity, which can catalyze hydrogen peroxide and regulate the level of reactive oxygen species (ROS) in the body, to achieve efficient sterilization and removal of bacterial biofilm [105]. Gao’s group focused on the iron–sulfur protein system, a class of proteins that affect the body’s redox homeostasis by regulating the amount of sulfur, by converting organic sulfur compounds of garlic into inorganic nano-iron sulfides, in which the antibacterial activity improved more than 500 times (Figure 3) [106]. Differently, the antibacterial mechanism of iron sulfides mainly includes the following two aspects, dissimilar to iron oxides: (1) Nano-iron-based sulfides have enzyme-like activity, can catalyze H_2_O_2_ to produce free radicals ·OH, and can promote lipid peroxidation of the cell membrane. (2) Hydrogen sulfide (H_2_S) gas will be produced under weakly acidic conditions by iron-based sulfides. Bacteria can be killed by the release of hydrogen polysulfide, and this small inorganic molecule can quickly permeate through the bacteria and significantly increase the level of ROS and lipid oxidation, eventually leading to bacterial death. Together, the bacterial death induced by iron sulfides has typical iron death characteristics. Along with the significant inhibition of the activity of the complex in the bacterial respiratory chain, glutathione (GSH) will be oxidized into oxidized glutathione (GSSG), resulting in GSH depletion and lipid peroxidation of the cell membrane [107]. However, not all iron sulfides have antibacterial activity, among which Fe_3_S_4_ and Fe_7_S_8_ have good antibacterial activity and antibacterial selectivity to gram-negative bacteria, especially to Gardnerella, with a minimum inhibitory concentration of 7.8 μg/mL, with the antibacterial effect better than that of metronidazole [108]. A disadvantage of iron-based sulfides is that they are unstable in aqueous systems, so iron-based sulfides such as FeS can also be encapsulated in acrylamide hydrogels, and the porous structure will not only capture and enrich bacteria, but also effectively retain active antibacterial substances, further enhancing antibacterial properties and wound healing [109]. The unique antibacterial properties and mechanisms of iron-based sulfides make them a potential novel class of non-antibiotic drugs for the prevention and treatment of bacterial vaginitis, dental caries and wound infection. 

### 4.2. Tumor Treatment

**Figure 4:**
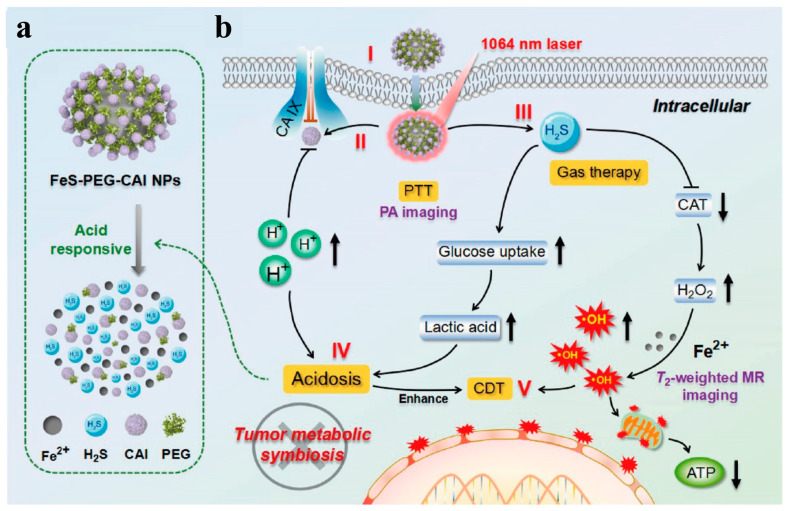
(**a**) Degradation behavior and (**b**) tumor therapy mechanism of FeS-PEG-CAI NPs [72]. Copyright 2022 American Chemical Society.

There has been a rapid revolution in the growing treatment of cancer, where some new treatments with low side effects and high specificity have been developed, including photothermal therapy [110], chemodynamic therapy [111], gas therapy [112], starvation therapy [113], etc. At the same time, cancer therapy is also expanding to multi-modal treatment. Inorganic nano-platforms are endowed with an intelligent response to special microenvironments, and they are easy to combine with exogenous physical tools (such as light, ultrasound, X-rays, magnetic fields, heat and electricity) to orthotopically activate the immune and other functions, leading to the implementation of more accurate and efficient cancer treatment. In the previous studies, iron-based sulfides have been explored that not only possess chemodynamic properties based on the Fenton reaction but also exhibit higher absorbance in the near infrared region (NIR), and this indicates iron-based sulfides can convert more absorbed NIR light into heart. This is why iron-based sulfides have potential applications in tumor treatment, including chemotherapy and photothermal therapy. For example, Zhang’s group presented an albumin-constrained strategy to synthesize tiny and highly dispersible ferrous sulfide (termed FeS@BSA) quantum dots at ambient conditions. FeS@BSA quantum dots have specific and strong absorption in the near infrared region, the temperature of FeS@BSA solution (1.2 mg/mL) rapidly increased up to 56.8 °C after being irradiated for 5 min by a 660 nm laser (2 W/cm^2^), which indicated the excellent photothermal conversion effect of FeS@BSA quantum dots [73]. The photothermal therapeutic effects of other iron-based sulfides on tumors are shown in Table 3. Later, Hou’s group reported a strategy based on carbonic anhydrase inhibitor (CAI)-modified ferrous sulfide nanoparticles (FeS-PEG-CAI NPs) that eliminate tumors by inducing acidosis to destroy the metabolic comorbidities within the tumor site, achieving PAI and MRI dual-mode guided chemotherapy/photothermal/gas therapy (Figure 4) [72]. If the size of FeS is controlled within the ultrasmall range, it will possess renal clearance properties, enhancing the potential for clinical transformation [73]. FeS was also found to be a drug-carrying nano-platform to load the anti-cancer drug Dox for multi-modal breast cancer treatment [114]. Yan’s group developed self-cascade pyrite nanozymes according to FeS_2_, contributing to apoptosis–ferroptosis synergistic tumor therapy. On top of that, they explained that when FeS_2_ interacts with the substrate H_2_O_2_, the ligand binding covalent bond is shorter than Fe_3_O_4_ nanoparticles. The surface exhibits many furl-like structures, conferring FeS_2_ greater binding competence with the substrate, resulting in the catalytic efficiency of H_2_O_2_ (K_cat_/K_m_) being 4144 times higher than that of traditional Fe_3_O_4_ nanoparticles, and 3086 times higher than that of natural horseradish peroxidase [115]. 

In general, the advantages of iron-based sulfides are based on the following aspects: (1) They can consume the endogenous H_2_O_2_ of tumor cells through the Fenton reaction to produce ROS with high cytotoxicity, which will further induce the apoptosis of tumor cells. (2) Iron-based sulfides produce and release H_2_S under weakly acidic conditions, which has a specific inhibitory effect on the catalase activity of cancer cells, and then increases the content of H_2_O_2_ in tumor cells, helping to enhance the CDT performance simultaneously. (3) Due to the strong optical absorption of iron sulfides in the near-infrared region, they can be used in the photothermal therapy of tumors based on the photothermal effect. (4) In particular, some iron-based sulfides can be seen as the initiators of ferroptosis that cause the inactivation of intracellular glutathione peroxidase 4 (GPX4) directly or indirectly, which leads to the collapse of the intracellular antioxidant system and, ultimately, cell death. At present, the difficulty is to overcome some universal problems such as the poor pharmacokinetics, low accumulation level of the target site and low biocompatibility, striving for the clinical transformation of iron-based sulfides.

### 4.3. Bio-Sensing

**Figure 5:**
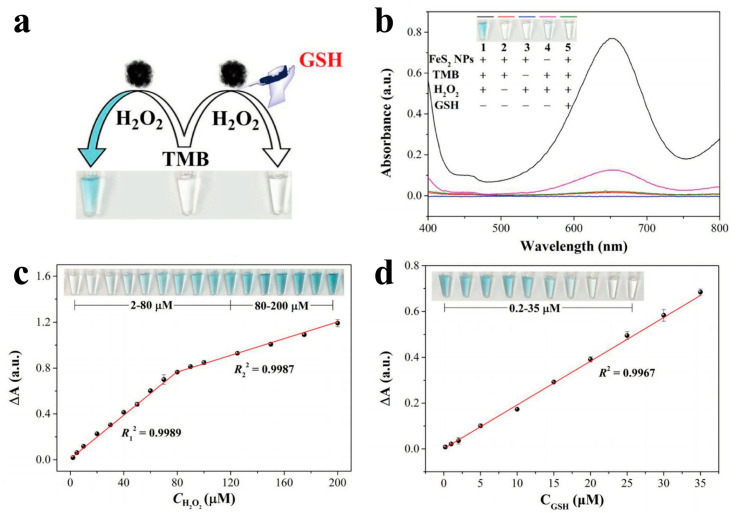
(**a**) Schematic illustration of colorimetric detection with H_2_O_2_ and GSH based on FeS_2_ NPs as the sensing platforms; (**b**) UV–Vis absorption spectra of the reaction system with different reagents; linear calibration plots for the detection of (**c**) H_2_O_2_ and (**d**) GSH. Inset: photographs of corresponding samples [117]. Copyright 2020 Elsevier.

Hydrogen peroxide (H_2_O_2_) and glutathione (GSH) are important molecules in life processes, which can be generally used as key biomarkers and prognostic indicators in biomedical diagnosis. Iron-based sulfides have peroxidase catalytic activity due to the electron transfer between Fe^2+^ and H_2_O_2_, which can be used to detect H_2_O_2_, GSH and other bioactive substances [116]. Therefore, they have the potential to be used to construct biosensors with high sensitivity, a low detection limit and a wide detection range. FeS_2_ nanoparticles have been shown to have high peroxidase catalytic activity, which can efficiently catalyze H_2_O_2_ to produce ·OH. When 3,3′,5,5′-tetramethylbenzidine (TMB) is used as the indicator of ·OH, the reaction system will change from colorless to blue, and there is a prominent absorption peak (652 nm) in the UV–Vis spectrum. When GSH is added, the blue color fades, and the absorption peak disappears (Figure 5). The detection limits of H_2_O_2_ and GSH are as low as 0.91 μM and 0.15 μM based on this colorimetric biosensor [117]. Huang et al. constructed FeS_2_/SiO_2_ mesoporous hollow spheres with higher activity on this basis. The Mi constant (K_m_) is 18 times smaller than that of FeS_2_ nanoparticles, and the catalytic efficiency is 16 times that of FeS_2_ nanoparticles. The response to H_2_O_2_ and GSH at room temperature takes only 1 min, which provides a material base for the construction of biosensors with high sensitivity and a rapid response [118]. 

### 4.4. Neurodegenerative Diseases

Neurodegenerative diseases, including Alzheimer’s disease, Parkinson’s disease and multiple sclerosis, are common in the elderly. Previously, it has been noted that iron disorders are linked to various neurodegenerative diseases, such as Parkinson’s disease and multiple sclerosis. To be specific, these brain regions are typically occupied by iron-rich microglia cells, which can induce an iron overload followed by a significant change in the transcriptional state of microglia, which will lead to the threat of ferroptosis [119]. Iatrogenic iron in metal implants is a potential risk factor for developing neurological diseases, along with a higher incidence of Parkinson’s disease. In the face of an attack of excess iron, glial cells act as neuroprotectors to accumulate more extracellular iron by upregulating divalentmetaltransporter 1 (DMT1), while neurons limit iron uptake by increasing DMT1 degradation [120]. However, over time, the microglia become overwhelmed and die, releasing stored iron ions and causing neurons to die together, which will further induce neurodegenerative disease [103]. These findings suggest that ferroptosis may be an essential mechanism of neuronal loss in neurodegenerative diseases; therefore, inhibiting neuronal iron death may interfere with the progression of neurodegenerative diseases. Ferroptosis suppressor protein 1 (FSP1), calcium-independent phospholipase iPLA2β, ubiquinol, glutathione peroxidase 4 (GPX4), NFS1 cysteine desulfurase, frataxin (FXN) and iron sulfur cluster assembly enzyme (ISCU) are all critical regulators of ferroptosis in the extrinsic or intrinsic pathway [121,122,123,124,125,126,127]. Among them, NFS1, FXN and ISCU play an essential role in producing iron sulfide clusters. Specifically, NFS1 is a rate-limiting enzyme in the biosynthesis of iron sulfide clusters, mainly providing sulfide and catalyzing the formation of iron sulfide clusters [128]. FXN serves as a transporter, delivering iron to the ISCU scaffold protein, where the final assembly is completed [129,130,131]. These results provide evidence for a way to treat neurodegenerative diseases by adjusting ferroptosis and controlling the synthesis of iron sulfide clusters, including intracellular iron uptake, iron utilization and conversion, and enzyme catalysis.

## 5. Conclusions

Iron-based sulfides have a variety of structures with multi-functionality, and good application potential in the biomedical field, including in the antibacterial field, tumor treatment, bioimaging, biosensing and the treatment of neurodegenerative diseases. In addition to their excellent optical, electrical, magnetic and thermal properties, the primary reasons why they can be widely used in the biomedical field are their biosafety and universal applicability. Although iron-based sulfides are effective materials in electrocatalysis and water pollution treatment, their research in the biomedical field is still in the early stages. Over the past decade, considerable progress has been achieved regarding their catalytic properties, magnetic properties and ferroptosis regulation. However, controlled synthesis is still in its infancy, and there is still a long way to go to resolve the dilemma regarding the biological mechanisms of iron sulfide clusters. In addition, if the clinical application prospects of iron-based sulfides are to be realized, it is also necessary to explore the suitable dosages for different diseases. Whereas numerous studies have shown that the iron balance is crucial for health, iron overload has been revealed to be linked to the pathogenesis of diseases such as Parkinson’s and Alzheimer’s. Therefore, the biological metabolic pathways and potential side effects of iron-based sulfides urgently need to be researched. It is highly likely that, in the future, iron-based sulfides will be able to serve as clinical drug candidates and show potential in novel biomedical fields such as self-assembling microrobots [132], tissue engineering [133], and biomimetic protein [134].

## Figures and Tables

**Table 1:** The crystal structure of common iron sulfide minerals.

Minerals	Main Component	Crystallographic System	Reference
Pyrite	FeS_2_	Cubic system	[30]
Pyrrhotite	Fe_1-x_S	Hexagonal or monoclinic system	[35]
Marcasite	FeS_2_	Orthogonal system	[36]
Mackinawite	FeS	Tetragonal system	[38,39]

**Table 2:** Magnetic properties of various iron-based sulfides.

Name	R_1_ (mM^−1^ S^−1^)	R_2_ (mM^−1^ S^−1^)	Morphology	Reference
FeS_2_	/	85.36	Nanodots	[69]
	/	31.836	Nanoparticles	[70]
	1	18.14	Nanocrystals	[71]
FeS	/	209.8	Nanoplates	[64]
	/	40.159	Nanoparticles	[72]
	5.35	/	Quantum dots	[73]
Fe_1-x_S	/	36.09	Nanocrystals	[74]

**Table 3:** Effect of photothermal therapy mediated by various iron-based sulfides.

Name	Laser	Photothermal Conversion Efficiency	Weight Extinction Coefficient (L g^−1^ cm^−1^)	Tumor Type	Reference
BSO-FeS_2_	808 nm	49.5%	/	4T1 cells	[59]
FeS_2_@BSA-Ce6	808 nm	/	/	4T1 cells	[69]
FeS_2_-350	915 nm	33.1%	/	7721 cells	[70]
FeS_2_-PEG	808 nm	28.6%	/	4T1 cells	[71]
FeS@BSA	660 nm	30.04%	/	4T1 cells	[73]
FeS-PEG	808 nm	/	15.5	4T1 cells	[64]
FeS-PEG-CAI	1064 nm	56.51%	/	4T1 cells	[72]
Fe_1-x_S-PVP	808 nm	24%	/	PAN-02 cells	[13]

## Data Availability

Not applicable.
